# Circumareolar Mastopexy with Multiple Glandular Plications for Symmetry of the Contra-Lateral Breast, in Patients Undergoing Breast Reconstruction with Prosthesis. Experience on 50 Cases

**DOI:** 10.4137/bcbcr.s826

**Published:** 2008-05-29

**Authors:** M. P. Serra, P. Longhi, E. Robotti

**Affiliations:** Ospedali Riuniti Bergamo, Bergamo, Italy

**Keywords:** breast reconstruction with prosthesis, symmetrization, mastopexy

## Abstract

4 years experience on 50 cases using the Elliott’s technique for symmetrization of the contra-lateral breast in patients undergoing breast reconstruction with an anatomical prosthesis is presented in this paper.

The Elliott’s technique with its double superior and horizontal plication is a suitable and long-lasting procedure for patients with small-moderate ptotic breast and elastic skin, who wish to have a simple procedure and an immediate result with minimal scars.

## Introduction

The most important target for the techniques of mastopexy is try to achieve a good symmetry with a natural shape, a fullness of the upper pole and a long-lasting result.

It is especially difficult to achieve this outcome when using a mastopexy procedure on the contra-lateral breast of patients who underwent a breast reconstruction with prosthesis, following a mastectomy for a cancer.

In fact the difference in the profile of the upper pole of the breast with prosthesis on one side and the contra-lateral mastopexy on the other’s, is currently a challenge for the plastic surgeon, in obtaining the most natural result.

There is a variety of different morphologic features among the patients, such as skin condition, degree of ptosis and presence of asymmetry, which can compromise the outcome of the mastopexy procedure.

The surgeon should assess the skin’s elasticity during the preoperative examination. This is necessary to choose the right procedure to perform and predict the final result.

Actually the skin without ability to retract will be significantly detrimental to the success of the operation.

The degree of ptosis and the presence of asymmetry are also important characteristics to keep in mind before performing the mastopexy techniques and must be measured.

The traditional procedures of mastopexy tend to include all these criterions above-listed, but none focuses on the problem of the different profiles of the upper pole of the breasts when one is reconstructed with prosthesis and the other has a mastopexy, which is inadequate to duplicate the fullness of the upper pole, typical of the reconstructed breast with prosthesis.

Elliott’s technique solves this problem, since it consists of a circumareolar mastopexy with a double superior and horizontal plication of the glandular breast combined with a vertical inferior median plication.

## Materials and Methods

From January 2001 to January 2005, 50 contra-lateral mastopexy according to Elliott^1^, for symmetrization of a breast reconstructed with an anatomical prosthesis were carried out.

The anatomical prosthesis was inserted in a submuscular plane, after a long period of expansion with an expander. This period of expansion was 4 months minimum.

All these patients had an anatomical prosthesis with a medium height and projection, in order to maintain a good degree of fullness of the upper pole, as requested by our patients.

The average age was 45 years old and the Elliott’s procedure was performed in most of the patients at the same time of the replacement of the expander with a definitive prosthesis.

Patients with a significant amount of extra skin were not good candidates for the circumareolar mastopexy.

Therefore it was important to assess preoperatively the skin condition, degree of ptosis and asymmetry.

**photo 1.**
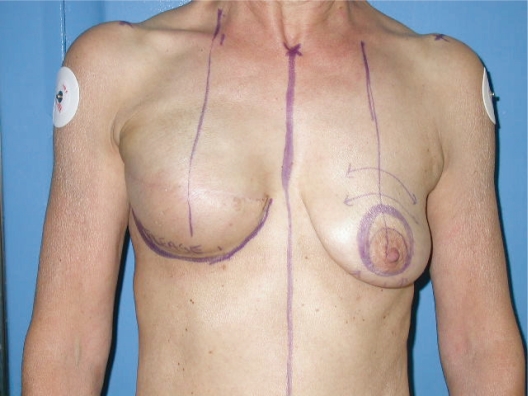
Preoperative marking.

Preoperative drawings were performed with the patient in a standing position (photo 1).

The measurements were made following the well known Elliott’s technique.

**Photo 2.**
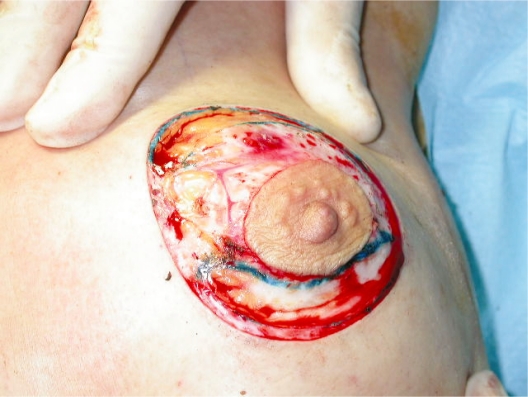
Incision of the dermis and degloving of the breast from 10 to 2 and 4 to 8 o’clock.

**Photo 3.**
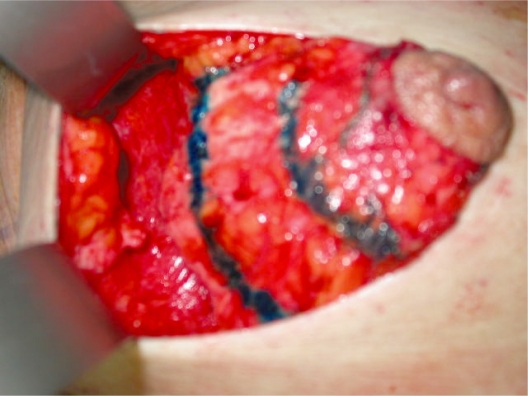
Double horizontal plications of the upper pole.

**Photo 4.**
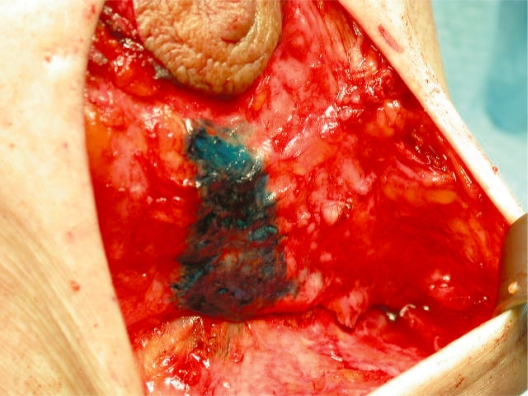
Vertical median plication of the lower pole.

The procedure consisted of:

1. circumareolar incision;
2. Almost complete degloving of the breast just deep to the skin from the 10 o’clock position to the 2 o’clock and from the 4 o’clock to the 8 o’clock position (photo 2).
3. Superiorly, plication of the breast tissue in tiers to itself, using absorbable sutures, each tier then stitched to the pectoralis major muscle: three sutures placed medially, three centrally and three laterally (photo 3).
4. Inferiorly, plication of the medial and lateral breast tissue to itself in the midline to give more projection to the nipple-areolar complex (photo 4).
5. Stitching of the circumareolar skin tightened around the nipple areolar complex, as described by Benelli.
6. Steri-strip applied over the suture lines and dressing to support the breast.

In all cases we didn’t use an implant on the mastopexy side.

## Results

This procedure has been used by the Authors for four years, with an average follow-up of 2 years.

The outcomes were related to the condition of the skin and degree of ptosis.

In our experience this technique appeared to solve the well known problem of inadequate projection of the upper pole of the contra-lateral breast, compared with the reconstructed breast.

The initially palpable superior glandular plications of the breast and the irregularity of the circumareolar scar, usually tended to solve in two months.

We revised 2 circumareolar scars for excessive rippling, because the suture broke few months after surgery.

Sometimes a short vertical scar had to be added either to reduce the periareolar tension or to remove the excess of inelastic skin and this was done in 4 patients with a 3rd degree of ptosis.

We had the best result in small and moderate ptotic breast, while severe ptosis often required the added of a vertical scar to decrease the postoperative rippling which did not disappear.

The procedure was safe and reliable.

The postoperative pain and recovery were reduced compared to the other techniques.

All patients were discharged between 2 and 4 days postoperatively and the drain on the side of the mastopexy was maintained for maximum 48 hours.

No haematoma or seroma were identified using this technique and no infection.

All these patients had an IV antibiotic prophylaxis 15–30 minutes before the induction, carried on for 48 hours and then continued with oral antibiotic for further 5 days.

The patient satisfaction was high, given that the expected result and the limitations of the technique were fully discussed with them preoperatively.

## Discussion

The traditional techniques of mastopexy tend mainly to reshape the breast tissue and reduce the excess of skin, while Elliott’s procedure focuses on solving the problem of the projection of the upper pole.

The Elliott’s procedure recreates an upper pole, initially overcorrected, then sufficiently full, in order to better simulate, like a prosthesis, the fullness of the upper pole of the reconstructed breast.

There are several techniques which try to achieve this outcome, all having in common the purpose of re-draping the breast tissue, without relying exclusively on skin remodeling.

Among these procedures, mastopexy with the employment of a glandular flap turned over and stitched to the pectoralis major muscle is a very complex technique, described by Lejour^2^.

Lejour’s technique is a difficult procedure which doesn’t produce an immediately aesthetically pleasing outcome.

Patients must accept the impossibility of achieving an immediate postoperative result and the need to wait for few months before seeing an improvement in their breast shape and contour.

The most long-lasting technique seems to be the Sampaio-Goes^3^ which use a mesh support system, but the use of this sheet can result in problems in detecting tumor in the ptotic breast.

For this reason we preferred to perform the Elliott’s technique in patients with a small-moderate ptotic breast, with good elasticity of the skin, since it is a safe and simple procedure, with an immediate long-lasting good cosmetic outcome.

We performed this technique on 50 patients with small and moderate ptotic breast, with a follow-up of two years.

All patients were satisfied with their results and we revised just 2 circumareolar scars, due to the excess of rippling.

We also tried to employ Elliott’s procedure in 4 patients with severe ptosis and inelastic skin. In these particular cases we found it necessary to add a short vertical scar to reduce the periareolar tension and remove the excess of inelastic skin, in order to obtain a good result.

The immediate cosmetic outcome was very satisfactory, and we also had a good symmetry between the breast reconstructed with antomical prosthesis and the contra-lateral breast having a mastopexy.

The main disadvantages which restrict and limit the use of this technique in severe ptosis are the following:

1. The decreased long-lasting outcome;
2. The need to add a short vertical scar;
3. The persistent postoperative rippling which rarely disappears;
4. The possibility of further mastopexy.

In our experience we can advise Elliott’s technique as a suitable and long-lasting procedure for patients with small-moderate ptotic breast and elastic skin, who require a simple technique and an immediate result with minimal scars.

## Figures and Tables

**Photo 5.**
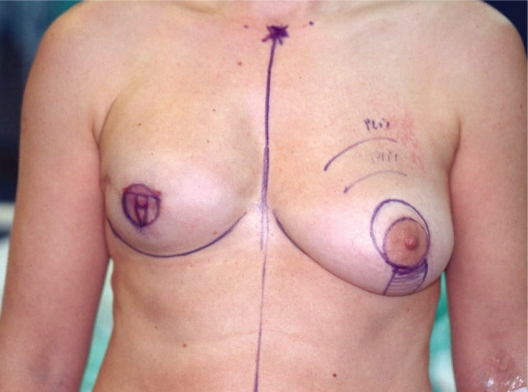
Right breast reconstruction with implant.

**Photo 6.**
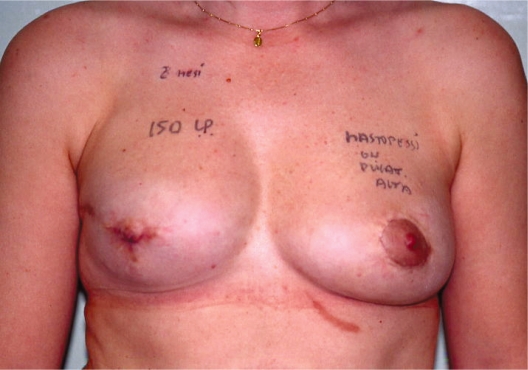
Right breast reconstruction with an anatomical prosthesis and contra-lateral mastopexy according to Elliott. Follow-up after 8 months.

**Photo 7.**
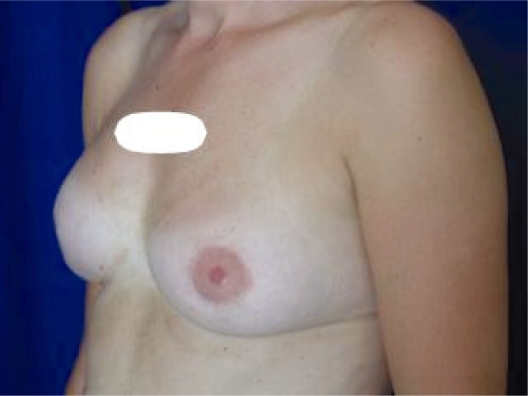
Left mastopexy. Follow-up after 2 years.

**Photo 8.**
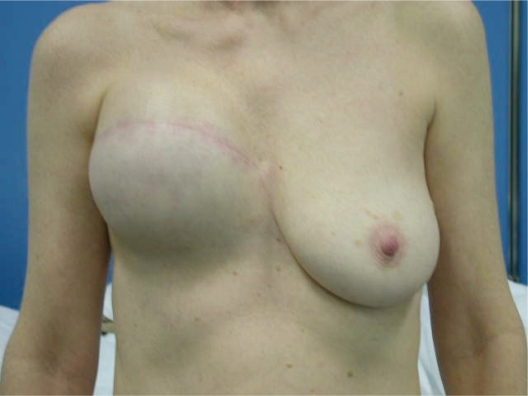
Expander right breast. Expansion after 4 months.

**Photo 9.**
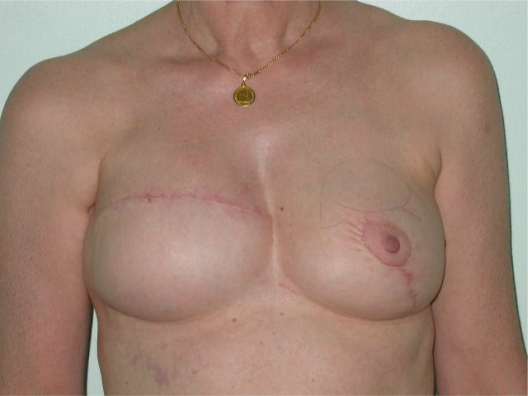
Right breast reconstruction with an anatomical prothesis and contra-lateral mastopexy according to Elliott. Follow-up after 1 year.
